# Transplantation: Polyomavirus Nephropathy and the Risk of Specific Immunosuppression Regimens

**DOI:** 10.1100/tsw.2006.93

**Published:** 2006-04-28

**Authors:** Christine Wu, Parmjeet Randhawa, Jerry McCauley

**Affiliations:** ^1^ Department of Medicine, Renal-Electrolyte Division, University of Pittsburgh, Pittsburgh, PA, USA; ^2^ 2Department of Pathology, Starzl Transplantation Institute, University of Pittsburgh, Pittsburgh, PA, USA

**Keywords:** polyomavirus, BK virus, BK nephropathy, kidney transplant

## Abstract

BK virus is ubiquitously present in the latent state in humans, and awareness of the importance of BK polyomavirus is emerging among the kidney transplant community. First discovered in 1971 in the urine of a renal transplant recipient, BK virus nephropathy (BKVN) has come to be recognized as a significant cause of genitourinary disease and potential graft loss in the kidney transplant patient. In this review, we discuss the risk factors, available methods of diagnosis and therapeutic monitoring, and current approaches to therapy of BKVN.

## BACKGROUND

Awareness of the importance of BK polyomavirus is emerging among the kidney transplant community. Polyomaviruses are biosafety level II, nonenveloped, icosahedral viruses measuring 40 nm in diameter, which consist of circular supercoiled dsDNA of 5 kb. There is an early region that encodes large T and small t proteins, a late region encoding the viral capsid, and a noncoding regulatory region. Polyomaviruses are highly species specific. The monkey simian virus (SV40) belongs to this family and is about 70% homologous with two polyomaviruses, BK and JC, which have been associated with human disease. BK virus (BKV) was first discovered in 1971 in the urine of a renal transplant recipient (with initials B.K.) who presented with ureteral stenosis. The patient's biopsy showed viral inclusions that did not stain for cytomegalovirus, herpes simplex virus, or adenovirus; electron microscopy confirmed viral particles, and antibody to SV40 classified the virus as a polyomavirus.

The precise mode of spread for BKV is unknown. There is no known animal reservoir. Primary infection occurs in early childhood (with peak age 2–5, as measured by antibody seroconversion) and is generally asymptomatic. In the U.S., 60–100% of adults exhibit serological evidence of past infection[1, 2,3]. In immunocompetent individuals, both BK and JC persist in the latent state predominantly in renal tubular and uroepithelial cells (but have also been found in B lymphocytes, leukocytes, liver, lung, and brain tissue) without causing disease[3,4], although occasional occurrences of cystitis have been reported[5]. In contrast, the virus can cause both symptomatic primary infection and reactivate in the immunocompromised state. Disease caused by BKV most commonly involves the genitourinary tract. BKV has been implicated in various tumors[6,7,8], although causality has yet to be proven, and its role in tumor development remains highly controversial. Fatal disease due to BKV infection has also been reported and includes death due to disseminated BKV in a child with hyper-IgM syndrome[9], interstitial pneumonia in an infant following hematopoietic stem-cell transplant[10], meningoencephalitis, retinitis and nephritis in AIDS patients[11], and death from endothelial infection with vasculopathy involving small vessels of cardiac and skeletal muscle after renal transplant[12].

In contrast to reports in the obstetrics and bone marrow transplant literature where disease is almost exclusively due to viral reactivation, approximately one-quarter of BKV infections in kidney transplant (KTX) recipients are primary infections[13]. Primary infection appears to occur early (10 days to 6 weeks), with the majority of disease from reactivation occurring later (5 weeks to 17 months)[14]. The median time to BKV nephritis is approximately 9–14 months post-transplant[15,16,17]. The majority of BKV nephropathy (BKVN) cases occur within the first postoperative year, but approximately one-quarter of cases present later[18]. However, the majority of cases of late rejection remain immunologic rather than viral in etiology.

Despite reports of extragenitourinary disease, BKV exhibits significant tropism to the genitourinary tract, which is the main site affected by BKV in the KTX patient. BKV can cause interstitial nephritis, ureteric stenosis, interstitial fibrosis, hemorrhagic cystitis, predisposition to secondary bacterial urinary tract infection[17], and graft loss[19]. At present, the KTX literature is lacking in prospective surveillance data of BK infection and is hampered by the lack of uniform detection and diagnosis methods. Therefore, the incidence of infection and BKVN is not well defined, but has been reported between 10–60%[19]. Reports of graft loss due to BKV has ranged from 1–5%[20,21] to greater than 80%[18,22,23,24,25], with perhaps lower rates in centers employing active surveillance and intervention protocols. The recent experience in our center since the institution of early diagnosis and therapy has led to maintenance of stable graft function for up to 2 years. Long-term graft survival is also felt to be worse in patients affected by BKV[17,26], although larger series are needed to determine the long-term prognosis of BKVN in the context of improved surveillance and earlier treatment. One large prospective cohort study demonstrated BKV infection in 65% of KTX recipients, but found no effect on mortality, graft function, or rate of graft loss[21].

The most common disorder caused by BKV in the KTX recipient is tubulointerstitial nephritis and is typically manifested by an increased creatinine, easily confused with rejection or drug toxicity. In fact, past diagnoses were often made after failed treatment of rejection[16]. Viral prodromal symptoms or the development of an echogenic renal mass concurrently with an increase in creatinine have been reported in some children with BKVN, but symptoms are rare in comparison to infections by other viruses such as cytomegalovirus (CMV) and Epstein Barr Virus (EBV)[27].

## RISK FACTORS

Multiple conditions have become generally accepted as risk factors for BKV infection, and the interaction between these factors may play as important a role as the presence of each individually.

Some controversy exists over the contribution of different drugs to the development of BKVN[26,28,29]. The existing literature consists of mainly small retrospective series. Cases of BKV infection began appearing in the literature in the 1990s, coinciding with the increased use of tacrolimus and cellcept, mycophenolate mofetil (MMF). In the existing published literature, over 90% of cases have been reported in patients exposed to tacrolimus or cellcept or both. Early reports demonstrated stabilization or improvement in disease with conversion from tacrolimus to cyclosporine[26,30]. However, this association does not establish causality and may reflect the prevalence of these two drugs in current immunosuppression regimens. In addition, the increasing prevalence of disease due to BKV is likely, in part, the result of bias due to enhanced surveillance and detection[31]. A retrospective review of biopsies from Basel, Switzerland from 1985–1995 reported the prevalence of BKV with the following immunosuppressive regimens: tacrolimus/cellcept 30/65(46%), cyclosporine/azathioprine 18/43 (41%), tacrolimus/azathioprine 19/69(27%), cyclosporine/cellcept 3/23 (13%)[25,32].

Antilymphocyte preparations for induction have not been significantly associated with BKVN, but their use in the treatment of rejection has been associated with virus replication in patients managed on, or switched to, triple therapy containing cellcept or tacrolimus[18,26]. The use of steroids for maintenance or treatment of rejection has been associated with an increased risk of BKVN, but only if maintenance immunosuppression was intensified. The risk of BKVN decreased, despite steroid use, if total maintenance immunosuppression was decreased[18,33]. In summary, the data remain unclear regarding the benefit of steroid avoidance or early tapering in regards to the development of BKVN[32].

The overall degree of immunosuppression may be a greater factor than the particular choice of any individual drug[34]. BKVN has been reported in a patient managed on sirolimus in the absence of either cellcept or tacrolimus[35]. Improvement or stabilization of disease has been reported in KTX recipients continued on reduced doses of cellcept or tacrolimus[36].

Other factors that increase the transplant patient's overall degree of immunosuppression have also been linked to an increased propensity toward development of BKVN and include frequency of treatment for acute cellular rejection (ACR) episodes and patient factors such as age[37,38], pharmacogenetics, cytokine profile (low interferon expression, high interleukin-6 [IL-6] or transforming growth factor-beta1 [TGF-β1] expression[39,40]), and comorbidities affecting immune status such as diabetes[17,38,41] or pregnancy[13]. BKV serostatus appears to be important, with the highest rates of disease occurring in seronegative pediatric recipients of seropositive donors[19,42,43]. Recipients of BKV seropositive donors with high viral loads also appear to be at increased risk for the development of BKVN[4,13,20].

Factors involving the microenvironment of the graft and its permissiveness likely play an important role as BKVN in the native kidney, while reported[44,45], is rare despite the use of many of the same medications in other solid organ transplant recipients. These factors include a history of graft injury, tissue ischemia[4,13,20], occurrence of rejection itself (separate from treatment of rejection)[46], and degree of antigen mismatch (HLA-ABDR)[47]. An increase in CD20 and decrease in cytotoxic T cells in the graft appears to be characteristic of BKVN[48]. Variables such as cold ischemia time, donor source (cadaveric vs. living), occurrence if delayed graft function have not been associated with BKVN[18,37,49].

Characteristics of the virus that may alter its pathogenicity also appear to be important. Genetic changes that alter the structure of the viral capsid protein (VP-1) or alter efficiency of gene expression of replication have been associated with disease[50,51]. Mutations in the VP-1 gene alter viral pathogenicity in mice. VP-1 sequence variations were noted in biopsies from patients with BKVN. Entry of the virus into the host cell is mediated by binding of VP-1 to an as yet uncharacterized protein containing α (2-3)-linked sialic residues and involves the caveolin pathway. Viral genomic regions relevant to pathogenesis of human disease include the noncoding control region (NCCR) that controls early and late transcription by enhancer elements, the T-antigen region, and the VP-1 region. NCCR rearrangement seen in cases of JC virus linked to progressive multifocal leukoencephalopathy does not appear to be requisite for the development of BKVN and were found in only 3/15 (20%) of patients with BKVN. In contrast, sequence alterations in transcription factor binding sites in NCCR are common in BKVN and included substitutions at Granulocyte Macrophage Stimulating Factor Promoter (22/26 biopsies), substitutions at NF-1 binding sites (4/24), duplicated AP-2 binding site (3/24). T-antigen mutations were commonly found, but produced amino acid changes in only 1/20 biopsies examined (glutamine to leucine). Unlike JC virus where pathogenicity is linked to viral genotype, genotypes did not appear to be altered in BKVN. Of 17 patients sequenced, 11 (65%) were found to be type 1, 1 (6%) type 2, 5 (29%) type 5, which is comparable to the type distribution in patients with asymptomatic viral shedding[52]. However, complete sequence identity with known genotypes was seen in only 5/49 biopsies. Temporal changes were also noted in all patients with multiple biopsies suggesting viral attempts at either evasion of host immunity or the development of drug resistance[50]. Other reported risk factors for BKVN include male gender and white ethnicity[37], and the placement of ureteral stents[25].

## DIAGNOSIS/ SCREENING/THERAPEUTIC MONITORING

The majority of adults harbor latent BKV and not all infection, whether primary or reactivation, leads to disease. In addition, not all disease leads to graft dysfunction. Therefore, the crux of BKV testing involves the ability to differentiate its benign presence from that of virus poised to damage the allograft, at an early enough stage where harm to the allograft is either preventable or reversible. Several urine, blood, and tissue tests are currently available. With the currently available noninvasive blood and urine assays, there remains considerable overlap of findings between asymptomatic, acute, and resolving BKVN. Also, the available assays have not been rigorously compared with each other in KTX recipients, and the sensitivity and specificities of urine and blood tests vary with a number of factors including the type of specimen being tested, the target sequences used for detection, the patient population under study, level of immunosuppression, and the timing of samples relative to diagnosis. Finally, as for all viral pathogens, absolute BK viral loads can differ significantly between laboratories[35], thus knowledge of a particular laboratory's operating characteristics remains crucial to the interpretation of results.

### Tissue Biopsy

The definitive diagnosis of BKVN is made by tissue biopsy. Histopathology also carries prognostic significance. The finding of tubular injury is most strongly correlated with the degree of allograft dysfunction[53]. The pathology of BKVN progresses through four phases: the first marked by silent viral inclusions initially involving the medulla (Fig. 1), the second by acute tubular necrosis (Fig. 2) possibly through a direct viral cytopathic effect, the third by viral interstitial nephritis with tubulitis (Fig. 3), and the fourth by chronic allograft nephropathy (Fig. 4). Thus, typical biopsy findings include severe tubular injury with mononuclear interstitial infiltrates, with or without tubulitis, epithelial necrosis, denuded tubular basement membrane, and focal intratubular neutrophilic infiltrates. These changes may be very focal with varying degrees of inflammatory cell infiltrates, tubular atrophy, and fibrosis[17,21,54,55,56].

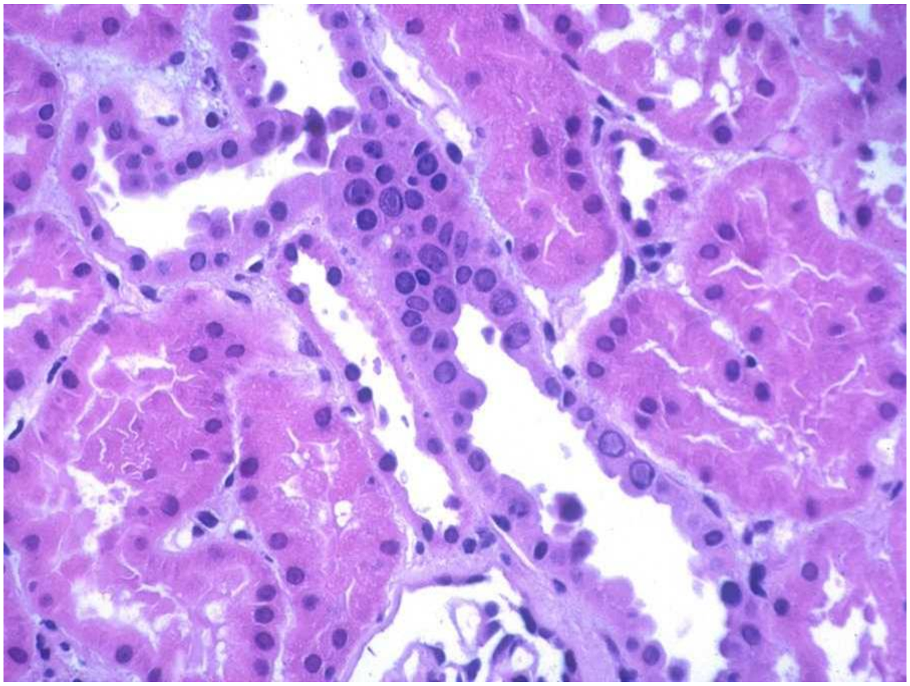

**FIGURE 1**. BKVN. H&E. Silent viral inclusions in medulla.

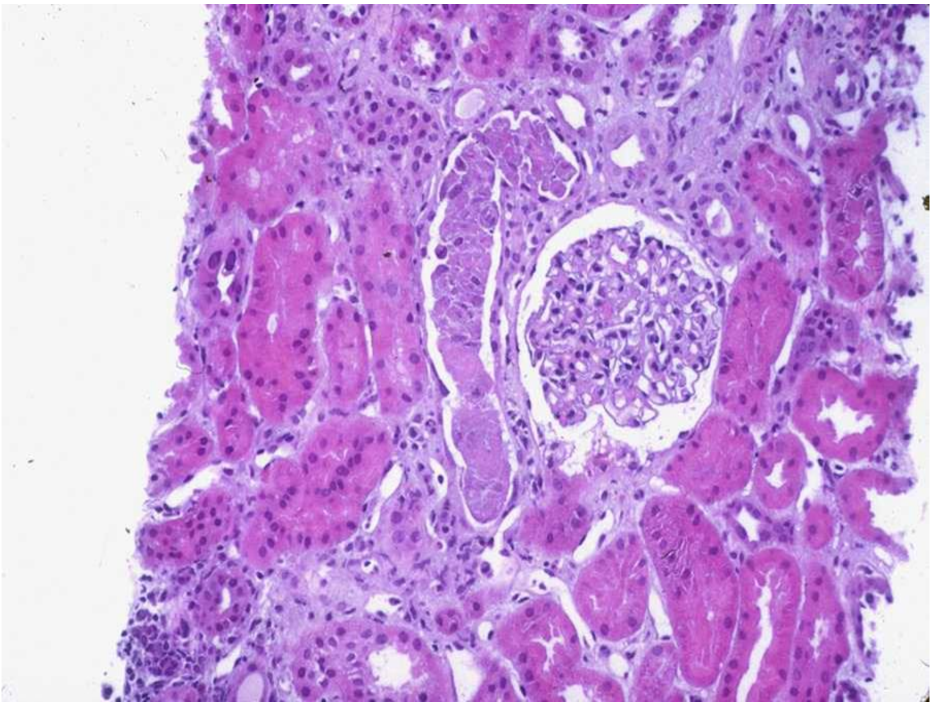

**FIGURE 2.** BKVN. H&E. Acute tubular necrosis.

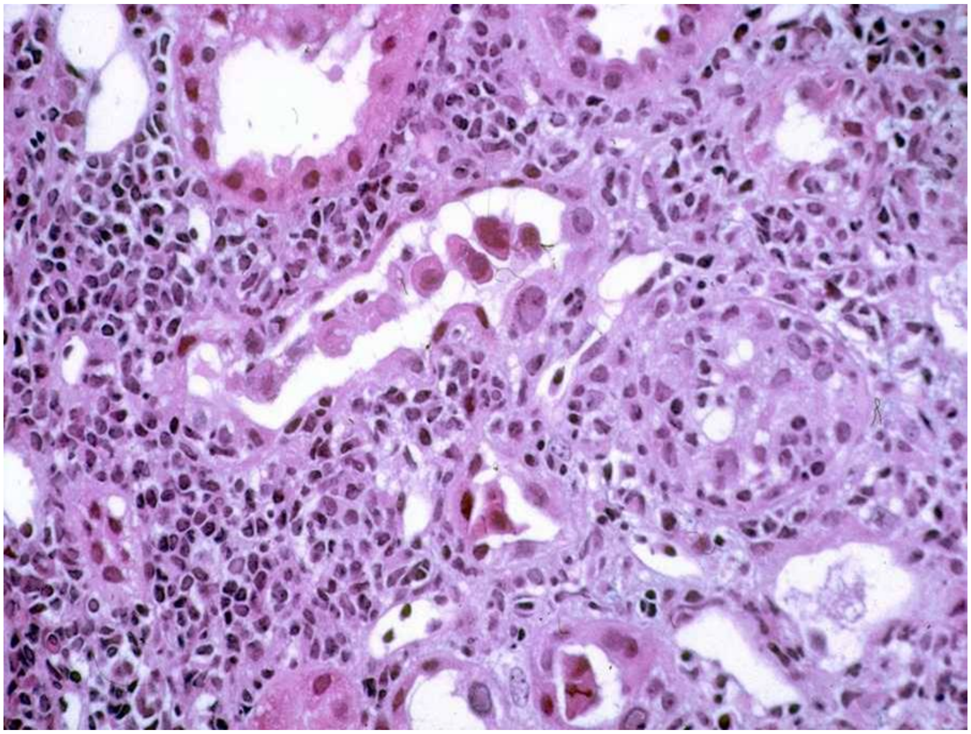

**FIGURE 3**. BKVN. H&E. Viral interstitial nephritis with tubulitis.

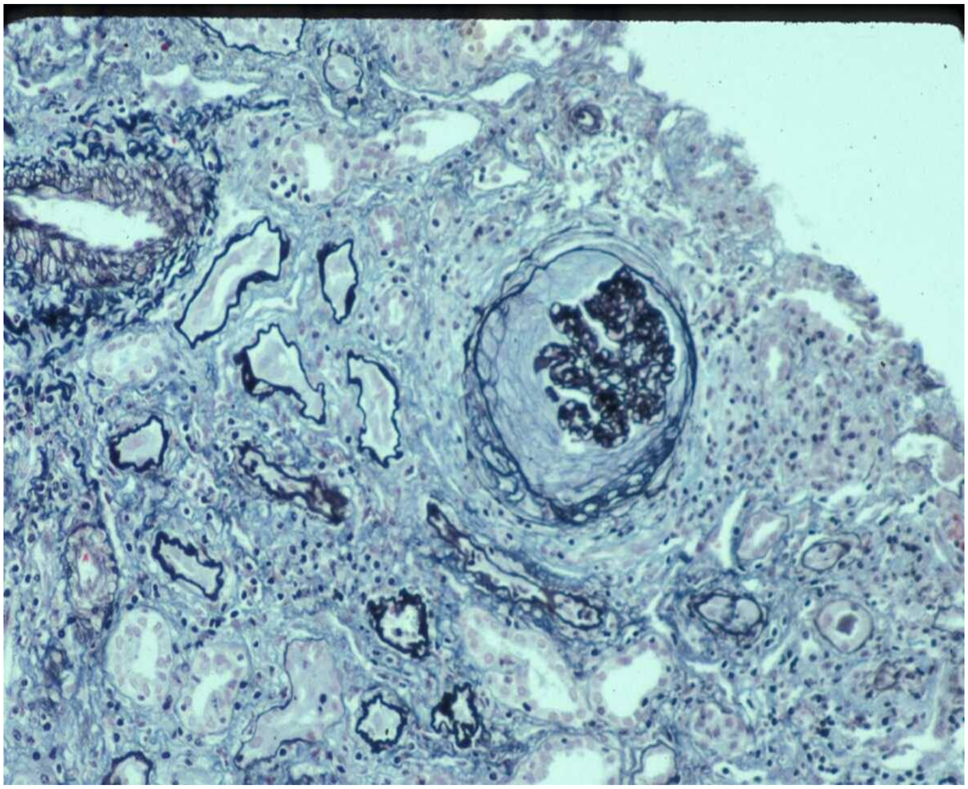

**FIGURE 4**. BKVN. Silver stain. Chronic allograft nephropathy.

Light microscopy is not pathognomonic. The biopsy appearance can be confused with rejection, even by experienced pathologists, and because the abnormalities can be focal, a negative biopsy cannot rule out disease[28]. Helpful findings include large tubular epithelial or glomerular parietal cells with nuclear atypia and basophilic or amphophilic intranuclear viral inclusions[17,28]. The presence of endarteritis, fibrinoid vascular necrosis, glomerulitis Banff type II and III, and peritubular capillary C4d deposition are generally considered to be more consistent with rejection[57,58]. Differentiating BKVN from cellular rejection with biopsy changes limited to Banff type I, which can be a manifestation of graft response to antigens in either the virus or the graft, remains difficult. Tubular HLA-DR expression, lymphocytic infiltrates, and tubulitis in areas without polyomavirus replication are suggestive of ACR[59].

For confirmation, most centers employ immunohistochemistry with antibodies to polyomavirus proteins. Some centers use *in situ* hybridization to identify polyomavirus genomes or electron microscopy to demonstrate virus particles measuring 45–55 nm in diameter[15,17,54,55] (Figs. 5,6,7). However, these tests are not specific for BKV. If light microscopy and immunohistochemistry are negative for findings of BKVN, but clinical suspicion is high and is supported by plasma or urine findings, repeat biopsy may be indicated.

Quantitative polymerase chain reaction (PCR) can also be performed on the biopsy specimen. One study found that patients with BKVN had a mean viral load of approximately 6000 copies/cell compared to controls with chronic allograft nephropathy, ACR, or CMV infection who had a mean viral load of 3.8 copies/cell. In the same study, a viral load of >59 copies/cell identified all cases of BKVN. Using this cut-off, their reported sensitivity, specificity, positive predictive value (PPV), and negative predictive value (NPV) were 100, 92.1, 73.6, and 100%, respectively. Lower levels were found in some specimens performed on patients who, on later biopsies, had a significant viral load and developed BKVN[60].

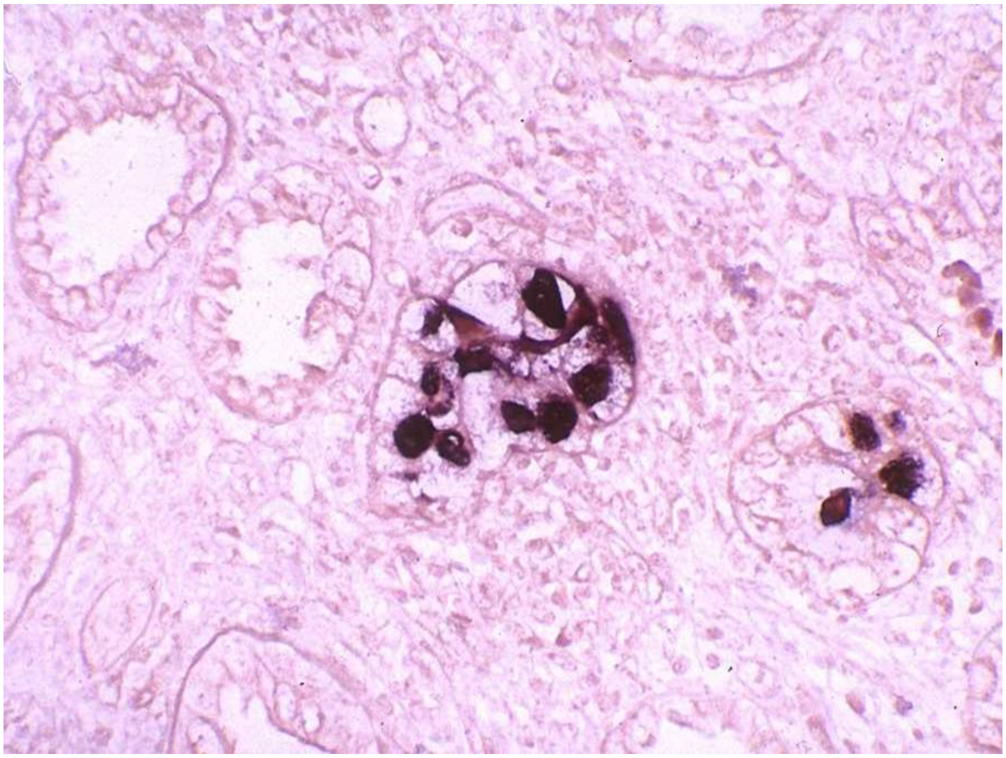

**FIGURE 5**. In-situ hybridization with a DNA probe to BKV genome showing infected tubular epithelium.

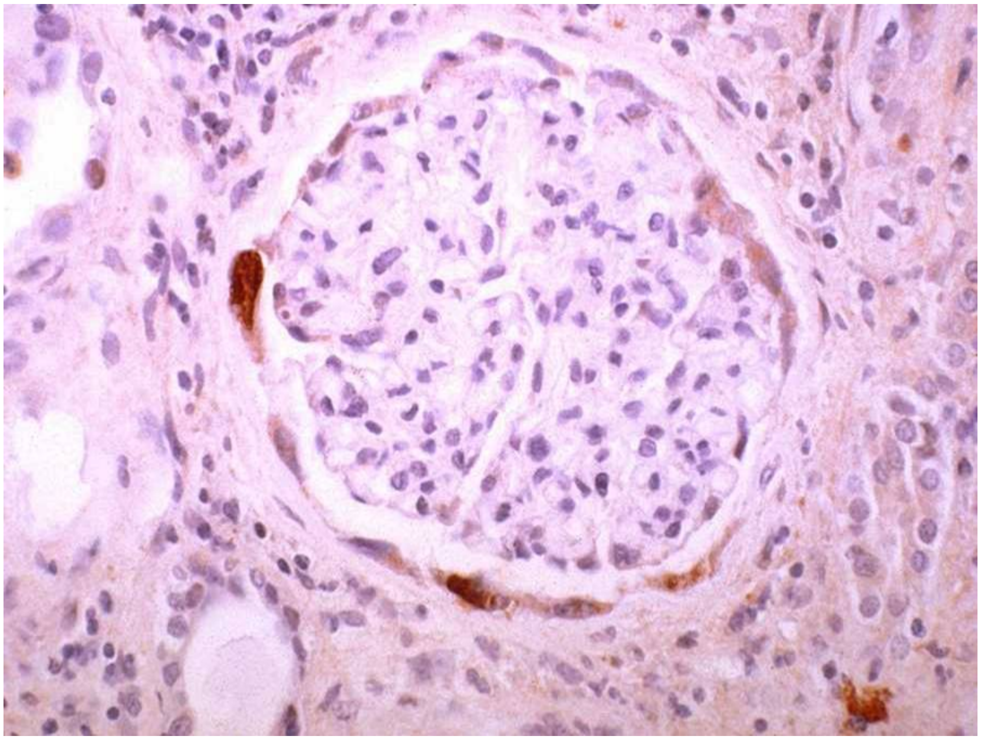

**FIGURE 6.** Immunohistochemistry with antibodies to polyomavirus antigens showing infected Bowman's capsular epithelium.

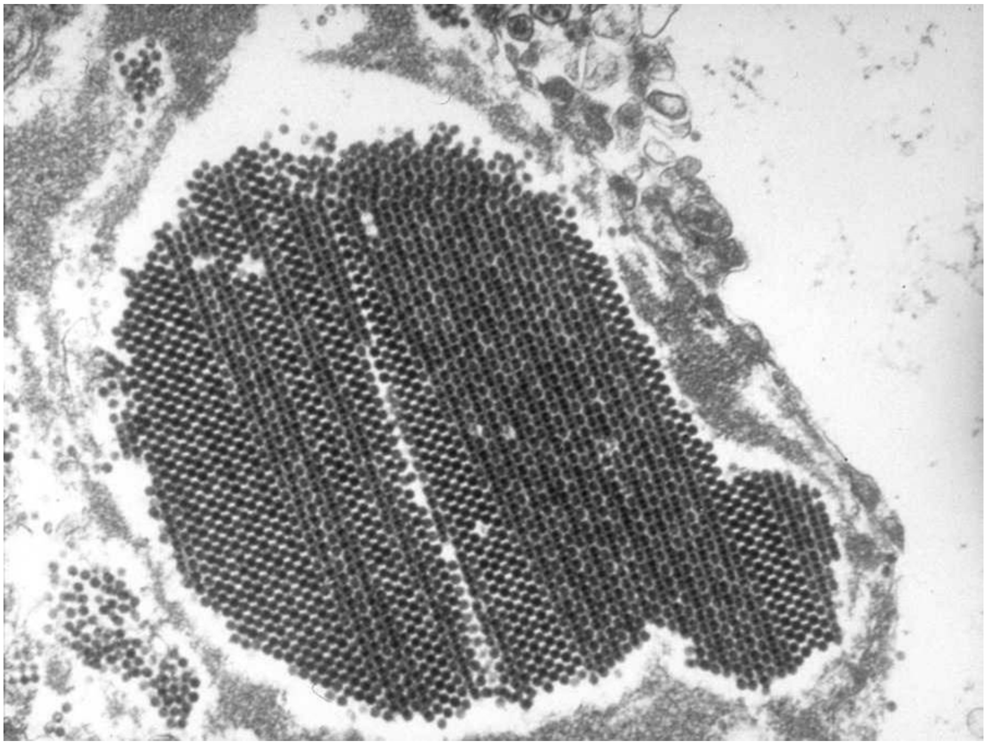

**FIGURE 7.** BKVN. Electron microscopy to demonstrate virus particles measuring 45–55 nm in diameter.

## Urine

Available urine studies include qualitative tests such as cell culture and cytology, and quantitative tests such as PCR. Cell culture is limited by low sensitivity, slow turnaround time (14–28 days), and subtle cytopathologic changes requiring an experienced interpreter. Infected cells shed into urine from renal tubules, particularly along the distal convoluted tubule and collecting duct, and superficial layers of the uroepithelium[15]. Cytology depends on the detection of these “decoy cells” in the urine, which are detected with the Papanicolaou stain, immunocytochemistry, or electron microscopy. Decoy cells have a characteristically enlarged nucleus with basophilic inclusions surrounded by ground-glass or clumped-appearing chromatin (Fig. 8). Viral inclusions can have a vesicular appearance or halo, and the cell body can appear neoplastic. CMV infection can have a similar appearance[61]. Immunocytochemistry helps to identify polyomavirus, however, it cannot specifically identify BKV from other polyomaviruses and only identifies viral antigen in abnormal cells. Results may not correlate with serology[62]. In addition, false-negative results can occur if the specimen is not sufficiently cellular, the virus load is low, or there is vaginal contamination. Sensitivity and NPV have been reported as approximately 40 and 80%, respectively[63]. There exists some disagreement on the predictive value of urine cytology as decoy cells may be present without clinical disease[21,54,56,59].

Quantitative PCR assays have been developed at various institutions. There is, as yet, no standard assay and thresholds for detection and significance vary between laboratories. Detection may also be affected by urine concentration. However, one study found quantitative real-time PCR results between labs to be equivalent within one log base 10[32]. Most laboratories use an arbitrary detection limit of 1000 DNA copies/ml. Higher-sensitivity PCR techniques that can detect fewer copies have been reported, but do not appear to enhance the predictive characteristics of the test[32]. Quantitative urine PCR has a reported sensitivity for BKVN of 100%, specificity of 95%, PPV of 84%, and NPV of 100%[36,64].

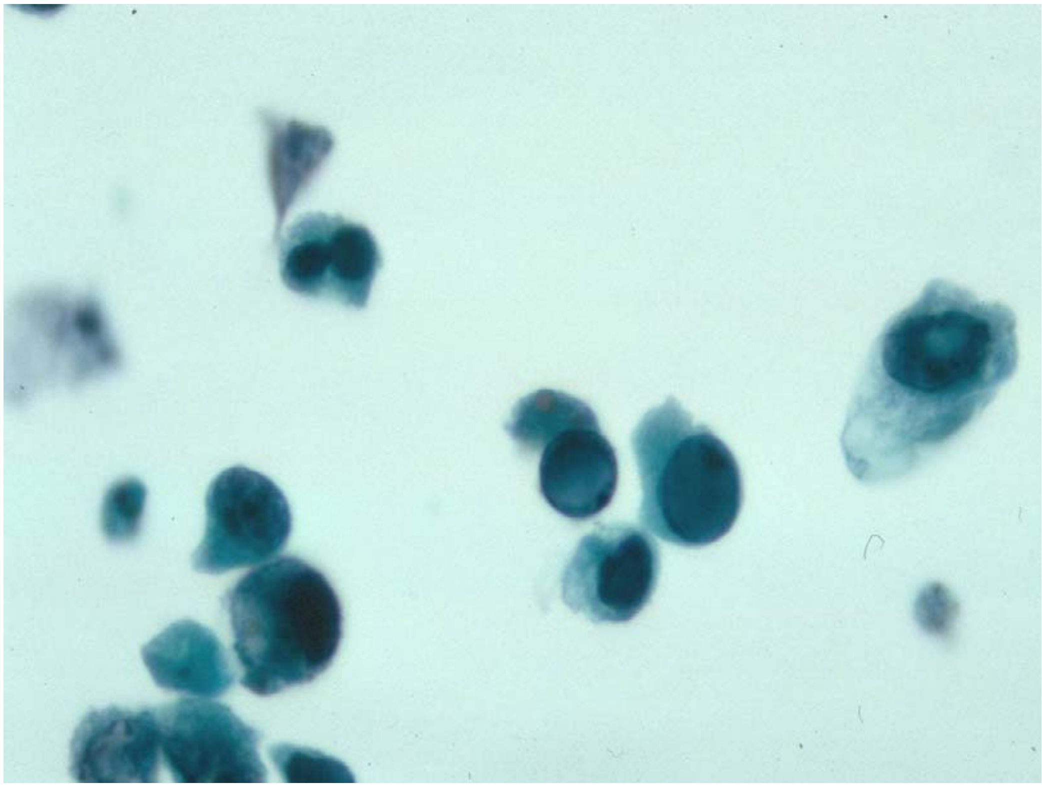

**FIGURE 8**. BKVN. Decoy cells in the urine. Papanicolaou stain.

Assays for 'BKV viral capsid protein 1 mRNA' have also been developed for urine. BKVN has been associated with urine VP-1 mRNA $\geq 6.5 \times 10^4$ copies/ng of total RNA yielding a sensitivity of 94% and specificity of 88%[24,32].

## Blood

A fourfold rise in antibody titer is suggestive of disease activation. Incomplete removal of nonspecific inhibitors can lead to false-positive results[65,66].

Quantitative PCR for BKV DNA can also be performed on peripheral blood samples. The thresholds for detection vary somewhat depending on the type of sample used for the DNA preparation (i.e., plasma, serum). The reported sensitivity and specificity for BKVN is reported as 100 and 88–95%, respectively, with a PPV of 82% and NPV of 100%[67]. Plasma viral load has been shown to fall after decreasing immunosuppression or following nephrectomy[68] and appears to closely follow the clinical course in BKVN, making it a useful test both for identifying patients at risk for the development of BKVN and for therapeutic monitoring[67,68].

## Recommendations:

To date, there is no large prospective study that demonstrates a consistent correlation between renal biopsy and a noninvasive assay. Biopsy remains the gold standard.

Findings and recommendations from the interdisciplinary work group convened in Basel, Switzerland in 2003 and published in *Transplantation* in 2005 are as follows[32]: Viuria by cytology or PCR appears to precede viremia by a median of 4 weeks and precede the appearance of histological abnormalities by a median of 12 weeks[18,25]. Screening for BKVN in urine and blood is recommended every 3 months during the first 2 years following kidney transplant and annually thereafter until the fifth post-transplant year. Testing is also recommended at any time a patient is evaluated for allograft dysfunction. Any positive screening test should be followed by confirmatory testing with biopsy within 4 weeks. Early biopsy based on surveillance has been associated with improved graft outcome[22].

In our laboratory, a urine PCR threshold of $\geq 1 \times e^7$ copies/ml in urine has been associated with a sensitivity of 100%, specificity of 96%, NPV of 100%, and PPV of 67% for the diagnosis of BKVN. A plasma threshold of 5000 copies/ml has yielded sensitivity of 100%, specificity 93%, NPV 100%, PPV 50%. Other laboratories have demonstrated a specificity for the detection of BKVN of ≥93% using a plasma threshold of 10,000 copies/ml[32,69]. Results persistently exceeding this threshold for greater than 3 weeks should be considered presumptive BKVN and should be followed by biopsy[32]. For the analysis of BKVN, the Banff working classification group has recommended the acquisition of at least two biopsy cores that contain medullary parenchyma[57]. Further research is needed to validate these recommendations.

Routine monitoring is obviously important to the early diagnosis of BKVN before significant graft injury occurs and to monitor response to titration of immunosuppression and antiviral therapy once the diagnosis is made. It may also play a future role in guiding preemptive therapy. There is currently insufficient evidence to guide management in this arena, however, for patients with persistently abnormal viral loads, but negative repeated biopsies, a preemptive decrease in immunosuppression could be considered. Once the diagnosis of BKVN is made, the patient should be monitored with quantitative urine and plasma studies every 2–4 weeks during therapy, with a therapeutic goal of achieving viral loads below the diagnostic threshold, and preferably below the level of detection[32]. Patients with resolved BKVN usually demonstrate clearance of viremia, decreased viuria, resolution of nuclear inclusions, and reversal of pathologic light microscopy findings, but persistent low-level DNA in biopsy specimens. If the patient fails to demonstrate a response to therapy, reassessment and repeat biopsy are recommended[32].

## THERAPY

### Decreasing Immunosuppression

In addition to the management of acute issues (i.e., hemodialysis for renal failure, surgical intervention for hydronephrosis or ureteral stenosis), the mainstay of current therapy remains the reduction of immunosuppression. No uniformly accepted recommendations for target drug levels or drug dosages exist. In addition, a KTX patient's overall state of immunosuppression is likely affected by factors other than medications such as age, comorbidity, and pharmacogenetics. One report did find that reducing tacrolimus trough levels from >9 to 6 ng/ml and cellcept doses to ≤1 g/day resulted in improvement or stabilization of disease in nine of ten patients studied[32].

Decreased immunosuppresion is associated with an increased risk of rejection, which has been reported in up to 25% of patients following dose adjustment[17,20,37]. However, the rejection episode may be steroid responsive, without recurrence[18,25]. Protocols for decreasing immunosuppression vary among institutions, with reported rates of graft loss ranging from <10% to >80%[17,20,31]. In the first year post-transplant, when the risk of rejection is highest, evidence exists to support switching or decreasing doses of medications rather than discontinuing a maintenance agent. Moderate evidence exists for substituting cyclosporine for tacrolimus, azathioprine for cellcept, and sirolimus for either tacrolimus or cellcept. Case reports have been published on the conversion from cellcept to leflunomide[70,71]. Evidence also supports decreasing goals for tacrolimus troughs to <6 ng/ml, cyclosporine troughs to 100–150 ng/ml, and cellcept doses to ≤1 g/day. Following the second post-transplant year when the risk of rejection diminishes, more centers are willing to consider discontinuing components of immunosuppression regimens (i.e., changing triple therapy to double), with regimens including cyclosporine and prednisone, tacrolimus and prednisone, sirolimus and prednisone, and cellcept and prednisone.

At the University of Pittsburgh, our first-line approach to BKVN is to reduce immunosuppression and consider antiviral therapy. Others have advocated in cases where BKVN occurs with concurrent ACR,, a two-step approach with treatment of rejection first, then reduced maintenance after 2 weeks is recommended[28,33,72,73]. The use of antilymphocyte preparations in the setting of BKVN is universally avoided, as their use in the treatment of rejection has been associated with increased virus replication in patients who were managed on, or subsequently switched to, triple therapy containing cellcept or tacrolimus[18,26]. Steroid use does not appear to worsen the overall course of BKVN if subsequent immunosuppression is decreased. Steroid use does not appear to be beneficial in the absence of rejection, and many centers avoid its use in the setting of active BKVN[74,75].

For patients considered to be too high risk to decrease immunosuppression (for example, patients with coexisting nonrenal solid organ transplants) or for those who have failed management with decreased immunosuppression alone, antiviral therapy may be indicated.

## Antiviral Therapy

Currently, cidofovir ([*S*]-9-(3-hydroxy-2-phosphonylmethoxypropyl)cytosine [HPMC]) is considered the most effective antiviral agent for the treatment of BKVN. Cidofovir is potentially effective for several DNA viruses, including herpesvirus, adenovirus, papillomavirus, polyomavirus, and poxvirus. It is FDA approved for the treatment of CMV retinitis in HIV-infected individuals. Despite only modest *in vitro* activity against BKV replication, cidofovir remains one of the most selective antipolyomavirus agents available[71,76]. Its first reported use for BKV infection was in a bone marrow transplant recipient treated for acute, severe hemorrhagic cystitis, resulting in symptomatic response after 2 weeks and persistent undetectable virus after greater than 5 months[77].

The major drawback to the use of cidofovir in the KTX population is its potential for nephrotoxicity. However, its selective concentration in renal tubular cells and urine supports the use of lower doses without probenicid in the setting of renal dysfunction. Recommendations for the off-label use of cidofovir for the treatment of BKVN include a dose of 0.25-1 mg/kg/dose intravenously every 2–3 weeks (10–20% of the current recommended dose to treat CMV retinitis) without probenicid, with enrollment in a clinical trial[32]. Four patients treated with low-dose cidofovir (0.25–1 mg /kg/dose every 2–3 weeks) without probenecid[27] achieved stable renal function after 6–26 months. Successful treatment has been reported separately in another two patients[78]. The results from a series of 26 patients in our institution, treated with low-dose cidofovir without probenicid, were recently reviewed [Scantlebury, V., Shapiro, R., Randhawa, P., Weck, K. (2002) *Graft*. **5**, S82-87.]. Decreased viruria within 4–12 weeks of treatment was demonstrated in all patients, with clearance of virus achieved in 25 of 26. Half of the patients exhibited improved graft function.

*In vitro* studies suggest that the beneficial effect of treatment is greatest when started early after the detection of infection[79]. Further studies are needed to refine the criteria for the usage, dosing, and monitoring of cidofovir and to evaluate its potential use in the prophylactic or preemptive treatment of BKV infection.

Leflunomide also has modest *in vitro* activity against BKV[71]. One study demonstrated decreased viremia and improved histology in 15 of 17 patients converted from cellcept to leflunomide[70]. Less success has been reported in other agents that have been tried for the management of BKVN. In a randomized, double-blind, placebo-controlled trial of 41 KTX patients, low-dose interferon prophylaxis did not reduce the rate of seroconversion or decrease viruria[80]. Use of vidarabine has been reported for the treatment of hemorrhagic cystitis in one bone marrow transplant recipient with resulting resolution of symptoms, but persistence of urine BKV DNA by PCR[81,82]. *In vitro* activity against BKV has been demonstrated with DNA gyrase inhibitors, retinoic acid, and 5-bromo-2'-deoxyurindine. A potential role for ciprofloxacin in the prevention of BKVN in patients undergoing allogeneic hematopoietic stem cell transplantation has been published. However, its modest *in vitro* activity casts doubt on its potential utility in the treatment of established BKVN.

## Retransplant

The first reported case of successful retransplantation following graft failure due to BKVN was reported in 2002[83]. The patient was originally managed on steroids, cellcept, and tacrolimus, received OKT3 for early ACR, but developed graft failure due to BKV interstitial nephritis despite decreased immunosuppression. He underwent an allograft nephrectomy, followed in 4 months by a living-related transplant, was managed postoperatively on tacrolimus, prednisone, and azathioprine, and had remained disease free for greater than 3 years. Surgical removal of the allloureter and graft could be considered in cases of persistent viral infection prior to retransplant, but the eradication of latent virus is not a realistic goal as it would necessitate removal of native kidneys and additional extrarenal sites harboring virus. In addition, the patient is likely to receive a seropositve graft. Recent reports have noted successful retransplant without allograft nephrectomy[84], and a recent review concluded that allograft nephrectomy is probably unnecessary[85]. The recurrence rate of BKVN has not yet been well established, but occurred in 2 of the first 15 patients to be retransplanted following known graft failure due to BKVN. The risk of recurrence, which may be higher following retransplant than after primary transplant, should be discussed with patients and any potential donors. Prior to retransplant, patients should be screened for BKV and immunosuppression minimized if possible. Recommendations for post-transplant BKV surveillance are currently the same as those for primary transplant. A history of graft failure due to BKVN is, therefore, not a contraindication to future transplant, and successful retransplant appears to be achievable if performed after the resolution of active lytic infection, with continued monitoring for prompt diagnosis and treatment, and avoidance of aggressive immunosuppression[86,87].

## CONCLUSION

- BKV ubiquitously present in latent state in humans.
- The spectrum of infection includes seroconversion, viruria, stricture, cystitis, and BKVN.
- Quantitative monitoring of viral load is useful for both screening patients and for monitoring Rx.
- Further development of safe and effective antivirals is needed to reduce the burden of disease in kidney transplant patients.

Recommended algorithm for the early diagnosis and management of BKVN.

## References

1. Sundsfjord, A., Spein, A.R., Lucht, E., Flaegstad, T., Seternes, O.M., and Traavik, T. (1994) Detection of BK virus DNA in nasopharyngeal aspirates from children with respiratory infections but not in saliva from immunodeficient and immunocompetent adult patients. *J. Clin. Microbiol.* **32,** 1390–1394.
2. Rziha, H.J., Bornkamm, G.W., and zur Hausen, H. (1978) BK virus: I. Seroepidemiologic studies and serologic response to viral infection. *Med. Microbiol. Immunol. (Berl.)* **165,** 73–81.
3. Reploeg, M.D., Storch, G.A., and Clifford, D.B. (2001) Bk virus: a clinical review. *Clin. Infect. Dis.* **33,** 191–202.
4. Mylonakis, E., Goes, N., Rubin, R.H., Cosimi, A.B., Colvin, R.B., and Fishman, J.A. (2001) BK virus in solid organ transplant recipients: an emerging syndrome. *Transplantation* **72,** 1587–1592.
5. Saitoh, K., Sugae, N., Koike, N., Akiyama, Y., Iwamura, Y., and Kimura, H. (1993) Diagnosis of childhood BK virus cystitis by electron microscopy and PCR. *J. Clin. Pathol.* **46,** 773–775.
6. Corallini, A., Pagnani, M., Viadana, P., Silini, E., Mottes, M., Milanesi, G., Gerna, G., Vettor, R., Trapella, G., Silvani, V., et al. (1987) Association of BK virus with human brain tumors and tumors of pancreatic islets. *Int. J. Cancer* **39,** 60–67.
7. Hogan, T.F., Padgett, B.L., Walker, D.L., Borden, E.C., and Frias, Z. (1983) Survey of human polyomavirus. *Prog. Clin. Biol. Res.* **105,** 311–324.
8. Geetha, D., Tong, B.C., Racusen, L., Markowitz, J.S., and Westra, W.H. (2002) Bladder carcinoma in a transplant recipient: evidence to implicate the BK human polyomavirus as a causal transforming agent. *Transplantation* **73,** 1933–1936.
9. Rosen, S., Harmon, W., Krensky, A.M., Edelson, P.J., Padgett, B.L., Grinnell, B.W., Rubino, M.J., and Walker, D.L. (1983) Tubulo-interstitial nephritis associated with polyomavirus (BK type) infection. *N. Engl. J. Med.* **308,** 1192–1196.
10. Sandler, E.S., Aquino, V.M., Goss-Shohet, E., Hinrichs, S., and Krisher, K. (1997) BK papova virus pneumonia following hematopoietic stem cell transplantation. *Bone Marrow Transplant.* **20,** 163–165.
11. Bratt, G., Hammarin, A.L., Grandien, M., Hedquist, B.G., Nennesmo, I., Sundelin, B., and Seregard, S. (1999) BK virus as the cause of meningoencephalitis, retinitis and nephritis in a patient with AIDS. *Aids* **13,** 1071–1075.
12. Petrogiannis-Haliotis, T., Sakoulas, G., Kirby, J., Koralnik, I.J., Dvorak, A.M., Monahan-Earley, R., De Girolami, P.C., De Girolami, U., Upton, M., Major, E.O., Pfister, L.A., and Joseph, J.T. (2001) BK-related polyomavirus vasculopathy in a renal-transplant recipient. *N. Engl. J. Med.* **345,** 1250–1255.
13. Andrews, C.A., Daniel, R.W., and Shah, K.V. (1983) Serologic studies of papovavirus infections in pregnant women and renal transplant recipients. *Prog. Clin. Biol. Res.* **105,** 133–141.
14. Gardner, S.D., MacKenzie, E.F., Smith, C., and Porter, A.A. (1984) Prospective study of the human polyomaviruses BK and JC and cytomegalovirus in renal transplant recipients. *J. Clin. Pathol.* **37,** 578–586.
15. Nickeleit, V., Hirsch, H.H., Binet, I.F., Gudat, F., Prince, O., Dalquen, P., Thiel, G., and Mihatsch, M.J. (1999) *J. Am. Soc. Nephrol.* **10,** 1080–1089.
16. Pappo, O., Demetris, A.J., Raikow, R.B., and Randhawa, P.S. (1996) Polyomavirus infection of renal allograft recipients: from latent infection to manifest disease. *Mod. Pathol.* **9,** 105–109.
17. Randhawa, P.S., Finkelstein, S., Scantlebury, V., Shapiro, R., Vivas, C., Jordan, M., Picken, M.M., and Demetris, A.J. (1999) Human polyoma virus infection of renal allografts: histopathologic diagnosis, clinical significance, and literature review. *Transplantation* **67,** 103–109.
18. Hirsch, H.H., Knowles, W., Dickenmann, M., Passweg, J., Klimkait, T., Mihatsch, M.J., and Steiger, J. (2002) Prospective study of polyomavirus type BK replication and nephropathy in renal-transplant recipients. *N. Engl. J. Med.* **347,** 488–496.
19. Shah, K.V. (2000) BKV and renal disease. *Nephrol. Dial. Transplant.* **15,** 754–755.
20. Randhawa, P.S. and Demetris, A.J. (2000) Nephropathy due to polyomavirus type BK. *N. Engl. J. Med.* **342,** 1361–1363.
21. Drachenberg, C.B., Beskow, C.O., Cangro, C.B., Bourquin, P.M., Simsir, A., Fink, J., Weir, M.R., Klassen, D.K., Bartlett, S.T., and Papadimitriou, J.C. (1999) Human polyoma virus in renal allograft biopsies: morphological findings and correlation with urine cytology. *Hum. Pathol.* **30,** 970–977.
22. Buehrig, C.K., Lager, D.J., Stegall, M.D., Kreps, M.A., Kremers, W.K., Gloor, J.M., Schwab, T.R., Velosa, J.A., Fidler, M.E., Larson, T.S., and Griffin, M.D. (2003) Influence of surveillance renal allograft biopsy on diagnosis and prognosis of polyomavirus-associated nephropathy. *Kidney Int.* **64,** 665–673.
23. Drachenberg, C.B., Papadimitriou, J.C., Wali, R., Nogueira, J., Mendley, S., Hirsch, H.H., Cangro, C.B., Klassen, D.K., Weir, M.R., Bartlett, S.T., and Ramos, E. (2004) Improved outcome of polyoma virus allograft nephropathy with early biopsy. *Transplant. Proc.* **36,** 758–759.
24. Ding, R. and Suthanthiran, M. (2003) Noninvasive diagnosis of BK virus nephritis by measurement of messenger RNA for BK VP1 virus in urine. *Transplantation* **76,** 446.
25. Brennan, D.C., Agha, I., Bohl, D.L., Schnitzler, M.A., Hardinger, K.L., Lockwood, M., Torrence, S., Schuessler, R., Roby, T., Gaudreault-Keener, M., and Storch, G.A. (2005) Incidence of BK with tacrolimus versus cyclosporine and impact of preemptive immunosuppression reduction. *Am. J. Transplant.* **5,** 582–594.
26. Binet, I., Nickeleit, V., Hirsch, H.H., Prince, O., Dalquen, P., Gudat, F., Mihatsch, M.J., and Thiel, G. (1999)

Polyomavirus disease under new immunosuppressive drugs: a cause of renal graft dysfunction and graft loss. *Transplantation* **67,** 918–922.
27. Vats, A., Shapiro, R., Singh Randhawa, P., Scantlebury, V., Tuzuner, A., Saxena, M., Moritz, M.L., Beattie, T.J., Gonwa, T., Green, M.D., and Ellis, D. (2003) Quantitative viral load monitoring and cidofovir therapy for the management of BK virus-associated nephropathy in children and adults. *Transplantation* **75,** 105–112.
28. Howell, D.N., Smith, S.R., Butterly, D.W., Klassen, P.S., Krigman, H.R., Burchette, J.L., Jr., and Miller, S.E. (1999) Diagnosis and management of BK polyomavirus interstitial nephritis in renal transplant recipients. *Transplantation* **68,** 1279–1288.
29. Barbanti-Brodano, G., Trabanelli, C., Lazzarin, L., Martini, F., Merlin, M., Calza, N., Corallini, A., and Tognon, M. (1998) [SV40 as a possible cofactor in the etiopathogenesis of mesothelioma and other human tumors]. *G. Ital. Med. Lav. Ergon.* **20,** 218–224.
30. Mathur, V.S., Olson, J.L., Darragh, T.M., and Yen, T.S. (1997) Polyomavirus-induced interstitial nephritis in two renal transplant recipients: case reports and review of the literature. *Am. J. Kidney Dis.* **29,** 754–758.
31. Hirsch, H.H. and Steiger, J. (2003) Polyomavirus BK. *Lancet Infect. Dis.* **3,** 611–623.
32. Hirsch, H.H., Brennan, D.C., Drachenberg, C.B., Ginevri, F., Gordon, J., Limaye, A.P., Mihatsch, M.J., Nickeleit, V., Ramos, E., Randhawa, P., Shapiro, R., Steiger, J., Suthanthiran, M., and Trofe, J. (2005) Polyomavirus-associated nephropathy in renal transplantation: interdisciplinary analyses and recommendations. *Transplantation* **79,** 1277–1286.
33. Celik, B., Shapiro, R., Vats, A., and Randhawa, P.S. (2003) Polyomavirus allograft nephropathy: sequential assessment of histologic viral load, tubulitis, and graft function following changes in immunosuppression. *Am. J. Transplant.* **3,** 1378–1382.
34. Andrews, C.A., Shah, K.V., Daniel, R.W., Hirsch, M.S., and Rubin, R.H. (1988) A serological investigation of BK virus and JC virus infections in recipients of renal allografts. *J. Infect. Dis.* **158,** 176–181.
35. Hirsch, H.H., Mohaupt, M., and Klimkait, T. (2001) Prospective monitoring of BK virus load after discontinuing sirolimus treatment in a renal transplant patient with BK virus nephropathy. *J. Infect. Dis.* **184,** 1494–1495; author reply 1495–1496.
36. Twu, J.S. and Schloemer, R.H. (1987) Transcriptional trans-activating function of hepatitis B virus. *J. Virol.* **61,** 3448–3453.
37. Ramos, E., Drachenberg, C.B., Portocarrero, M., et al. (2002) BK virus nephropathy diagnosis and treatment: experience at the University of Maryland Renal Transplant Program. *Clin Transpl.,* 143-153.
38. Trofe, J., Gaber, L.W., Stratta, R.J., et al. (2003) Polyomavirus in kidney and kidney-pancreas transplant recipients. *Transpl Infect Dis*. **5(1)**, 21-28.
39. Comoli, P., Azzi, A., Maccario, R., et al. (2004) Polyomavirus BK-specific immunity after kidney transplantation. *Transplantation* **78(8),** 1229-1232.
40. Voltz, R., Jager, G., Seelos, K., Fuhry, L., Hohlfeld, R. (1996) BK virus encephalitis in an immunocompetent patient. *Arch Neurol* **53(1)**, 101-103.
41. Hogan, T.F., Borden, E.C., McBain, J.A., Padgett, B.L., and Walker, D.L. (1980) Human polyomavirus infections with JC virus and BK virus in renal transplant patients. *Ann Intern Med* **92(3)**, 373-378.
42. Ginevri, F., De Santis, R., Comoli, P., et al. (2003) Polyomavirus BK infection in pediatric kidney-allograft recipients: a single-center analysis of incidence, risk factors, and novel therapeutic approaches. *Transplantation* **75(8),** 1266-1270.
43. Shah, K., Daniel, R., Madden, D., and Stagno, S. (1980) Serological investigation of BK papovavirus infection in pregnant women and their offspring. *Infect Immun* **30(1),** 29-35.
44. Limaye, A.P., Smith, K.D., Cook, L., et al. (2005) Polyomavirus nephropathy in native kidneys of non-renal transplant recipients. *Am J Transplant* **5(3)**, 614-620.
45. Hwang, E.A., Kang, M.J., Han, S.Y., Park, S.B., and Kim, H.C. (2004) Viral infection following kidney transplantation: long-term follow-up in a single center. *Transplant Proc* **36(7),** 2118-2119.
46. Atencio, I.A., Shadan, F.F., Zhou, X.J., Vaziri, N.D., and Villarreal, L.P. Adult mouse kidneys become permissive to acute polyomavirus infection and reactivate persistent infections in response to cellular damage and regeneration. *J Virol* **67(3)**, 1424-1432.
47. Awadalla, Y., Randhawa, P., Ruppert, K., Zeevi, A., and Duquesnoy, R.J. (2004) HLA mismatching increases the risk of BK virus nephropathy in renal transplant recipients. *Am J Transplant* **4(10)**, 1691-1696.
48. Ahuja, M., Cohen, E.P., Dayer, A.M., et al. (2001) Polyoma virus infection after renal transplantation. Use of immunostaining as a guide to diagnosis. *Transplantation* **71(7)**, 896-899.
49. Priftakis, P., Bogdanovic, G., Tyden, G., and Dalianis, T. (2000) Polyomaviruria in renal transplant patients is not correlated to the cold ischemia period or to rejection episodes. *J Clin Microbiol* **38(1),** 406-407.
50. Randhawa, P.S., Khaleel-Ur-Rehman, K., Swalsky, P.A., et al. (2002) DNA sequencing of viral capsid protein VP-1 region in patients with BK virus interstitial nephritis. *Transplantation* **73(7),** 1090-1094.
51. Randhawa, P., Zygmunt, D., Shapiro, R., et al. (2003) Viral regulatory region sequence variations in kidney tissue obtained from patients with BK virus nephropathy. *Kidney Int* **64(2),** 743-747.
52. Baksh, F.K., Finkelstein, S.D., Swalsky, P.A., Stoner, G.L., Ryschkewitsch, C.F., and Randhawa, P. (2001) Molecular genotyping of BK and JC viruses in human polyomavirus-associated interstitial nephritis after renal transplantation. *Am J Kidney Dis*, **38(2),** 354-365.
53. Nickeleit, V., Singh, H.K., and Mihatsch, M.J. (2003) Polyomavirus nephropathy: morphology, pathophysiology, and clinical management. *Curr Opin Nephrol Hypertens.*, **12(6)**, 599-605.
54. Drachenberg, R.C., Drachenberg, C.B., Papadimitriou, J.C., et al. (2001) Morphological spectrum of polyoma virus disease in renal allografts: diagnostic accuracy of urine cytology. *Am J Transplant* **1(4),** 373-381.
55. Drachenberg, C.B., Papadimitriou, J.C., Wali, R., Cubitt, C.L., and Ramos, E. (2003) BK polyoma virus allograft nephropathy: ultrastructural features from viral cell entry to lysis. *Am J Transplant* **3(11),** 1383-1392.
56. Drachenberg, C.B., Papadimitriou, J.C., Hirsch, H.H., et al. (2004) Histological patterns of polyomavirus nephropathy: correlation with graft outcome and viral load. *Am J Transplant* **4(12)**, 2082-2092.
57. Racusen, L.C., Rayner, D.C., Trpkov, K., Olsen, S., and Solez, K. (1995) The Banff classification of renal allograft pathology: where do we go from here? *Transplant Proc* **27(5),** 2561-2563.
58. Racusen, L.C., Colvin, R.B., Solez, K., et al. (2003) Antibody-mediated rejection criteria - an addition to the Banff 97 classification of renal allograft rejection. *Am J Transplant* **3(6),** 708-714.
59. Nickeleit, V., Hirsch, H.H., Zeiler, M., et al. (2000) BK-virus nephropathy in renal transplants-tubular necrosis, MHC-class II expression and rejection in a puzzling game. *Nephrol Dial Transplant* **15(3),** 324-332.
60. Randhawa, P.S., Vats, A., Zygmunt, D., et al. (2002) Quantitation of viral DNA in renal allograft tissue from patients with BK virus nephropathy. *Transplantation* **74(4)**, 485-488.
61. Fogazzi, G.B., Cantu, M., and Saglimbeni, L. (2001) 'Decoy cells' in the urine due to polyomavirus BK infection: easily seen by phase-contrast microscopy. *Nephrol Dial Transplant* **16(7),** 1496-1498.
62. Hogan, T.F., Padgett, B.L., Walker, D.L., Borden, E.C., and McBain, J.A. (1980) Rapid detection and identification of JC virus and BK virus in human urine by using immunofluorescence microscopy. *J Clin Microbiol* **11(2),** 178-183.
63. Randhawa, P., Vats, A., and Shapiro, R. Monitoring for polyomavirus BK And JC in urine: comparison of quantitative polymerase chain reaction with urine cytology. *Transplantation* **79(8),** 984-986.
64. Lin, P.L., Vats, A.N., and Green, M. (2001) BK virus infection in renal transplant recipients. *Pediatr Transplant* **5(6),** 398-405.
65. Flaegstad, T., Ronne, K., Filipe, A.R., and Traavik, T. (1989) Prevalence of anti BK virus antibody in Portugal and Norway. Scand J *Infect Dis* **21(2)**, 145-147.
66. Dei R, Marmo F, Corte D, Sampietro MG, Franceschini E, Urbano P. (1982) Age-related changes in the prevalence of precipitating antibodies to BK virus in infants and children. *J Med Microbiol* **15(3)**, 285-291.
67. Nickeleit, V., Klimkait, T., Binet, I.F., et al. (2000) Testing for polyomavirus type BK DNA in plasma to identify renal-allograft recipients with viral nephropathy. *N Engl J Med* **342(18)**, 1309-1315.
68. Limaye, A.P., Jerome, K.R., Kuhr, C.S., et al. (2001) Quantitation of BK virus load in serum for the diagnosis of BK virus-associated nephropathy in renal transplant recipients. *J Infect Dis* **183(11),** 1669-1672.
69. Randhawa, P., Ho, A., Shapiro, R., et al. (2004) Correlates of quantitative measurement of BK polyomavirus (BKV) DNA with clinical course of BKV infection in renal transplant patients. *J Clin Microbiol* **42(3),** 1176-80.
70. Williams, J.W., Javaid, B., Kadambi, P.V., et al. (2005) Leflunomide for polyomavirus type BK nephropathy. *N Engl J Med* **352(11)**,1157-1158.
71. Farasati, N.A., Shapiro, R., Vats, A., and Randhawa, P. (2005) Effect of leflunomide and cidofovir on replication of BK virus in an in vitro culture system. *Transplantation* **79(1),** 116-118.
72. Randhawa, P., and Shapiro, R. (2004) Conceptual problems in the diagnosis and therapy of acute rejection in patients with polyomavirus nephropathy. *Am J Transplant* **4(5)**, 840.
73. Mayr, M., Nickeleit, V., Hirsch, H.H., Dickenmann, M., Mihatsch, M.J., and Steiger J. (2001) Polyomavirus BK nephropathy in a kidney transplant recipient: critical issues of diagnosis and management. *Am J Kidney Dis* **38(3)** E13.
74. Purighalla, R., Shapiro, R., McCauley, J., and Randhawa, P. (1995) BK virus infection in a kidney allograft diagnosed by needle biopsy. *Am J Kidney Dis* **26(4),** 671-673.
75. Barri, Y.M., Ahmad, I., Ketel, B.L., et al. (2001) Polyoma viral infection in renal transplantation: the role of immunosuppressive therapy. *Clin Transplant* **15(4)**, 240-246.
76. De Clercq, E. (1997) In search of a selective antiviral chemotherapy. *Clin Microbiol Rev* **10(4)**, 674-693.
77. Gonzalez-Fraile, M.I., Canizo, C., Caballero, D., et al. (2001) Cidofovir treatment of human polyomavirus-associated acute haemorrhagic cystitis. *Transpl Infect Dis* **3(1),** 44-46.
78. Kadambi, P.V., Josephson, M.A., Williams, J., et al. (2003) Treatment of refractory BK virus-associated nephropathy with cidofovir. *Am J Transplant* **3(2)**, 186-191.
79. Andrei, G., Snoeck, R., Vandeputte, M., and De Clercq, E. (1997) Activities of various compounds against murine and primate polyomaviruses. *Antimicrob Agents Chemother* **41(3)**, 587-593.
80. Cheeseman, S.H., Black, P.H., Rubin, R.H., Cantell, K., and Hirsch, M.S. (1980) Interferon and BK Papovavirus--clinical and laboratory studies. *J Infect Dis* **141(2),** 157-161.
81. Vianelli, N., Renga, M., Azzi, A., et al. (2000) Sequential vidarabine infusion in the treatment of polyoma virus-associated acute haemorrhagic cystitis late after allogeneic bone marrow transplantation. *Bone Marrow Transplant* **25(3)**, 319-320.
82. Chapman, C., Flower, A.J., Durrant, S.T. (1991) The use of vidarabine in the treatment of human polyomavirus associated acute haemorrhagic cystitis. *Bone Marrow Transplant* **7(6)**., 481-483.

83. Poduval, R.D., Meehan, S.M., Woodle, E.S., et al. (2002) Successful retransplantation after renal allograft loss to polyoma virus interstitial nephritis. *Transplantation* **73(7)**, 1166-1169.
84. Ginevri, F., Pastorino, N., de Santis, R., et al. (2003) Retransplantation after kidney graft loss due to polyoma BK virus nephropathy: successful outcome without original allograft nephrectomy. *Am J Kidney Dis* **42(4)**, 821-825.
85. Ramos, E., Vincenti, F., Lu ,W.X, et al. (2004) Retransplantation in patients with graft loss caused by polyoma virus nephropathy. *Transplantation* **77(1)**, 131-133.
86. Leung, A., Chan, M., Yuen, K., Cheng, V., Chan, K., Wong, C., Liang, R., Lie, A., and Kwong, Y. (2005) Ciprofloxacin Decreased Polymoma BK Virus Load in Patients Who Underwent Allogeneic Hematopoietic Stem Cell Transplantation. *Clin. Inf. Dis.* **40**, 528-537.
87. Randhawa, P. (2005) Anti-BK Virus Activity of Ciprofloxacin and Related Antibiotics. (Letter) *Clin. Inf. Dis.*, **41**, 1366-1367.
